# Use of hybrid versus video-only teledermatology varies by condition: A cross-sectional study of 2064 encounters

**DOI:** 10.1016/j.jdin.2025.10.001

**Published:** 2025-10-16

**Authors:** Jacqueline Penn, Kathryn Achuck, Christine Deng, Richard Hass, Elizabeth Jones

**Affiliations:** a NYC Health and Hospitals, Bellevue, New York, New York; b Santa Clara Valley Medical Center, San Jose, California; c Drexel University, Philadelphia, Pennsylvania; d Thomas Jefferson University, Philadelphia, Pennsylvania

**Keywords:** general dermatology, hybrid dermatology, teledermatology, telemedicine, video dermatology

*To the Editor:* Teledermatology enhances the practice of dermatology by improving efficiency, cost-effectiveness, and access to care without compromising patient satisfaction.^1^, ^2^, ^3^ As medicine anticipates future adoption of technology in practice,^4^ tailoring the choice of the teledermatology model based upon the condition can improve practice implementation.

This is a retrospective cross-sectional single-institution study of 2984 synchronous teledermatology visits performed by 11 dermatologists using the Epic platform from March 14, 2020, through March 31, 2021. The visits’ first-listed International Classification of Disease (ICD-10) code was categorized by a board-certified dermatologist into 3 categories: acneiform, inflammatory, or lesion-specific. The first or index visit (*n* = 2064) was categorized as “hybrid,” if at least 1 photo was uploaded within 4 days before or after the encounter, or “video-only”. Two reviewers deemed photos unusable if the subject could not be adequately visualized.

Video-only visits comprised the majority of teledermatology visits (1797, 87%) as compared to hybrid visits (267, 23% with 827 photos). Inflammatory conditions were most often assessed (49.0%) followed by acneiform conditions (31%) and lesions (20%) (Table I). Chi-squared analysis revealed significant differences in use of hybrid versus video-only visits across the diagnostic categories (*P* < .001). Acneiform conditions were treated more frequently with video-only visits, while lesion-specific conditions were treated more frequently with hybrid visits. Inflammatory conditions were treated comparably with both models.

**Table I:** Number of visits by teledermatology (TD) model and primary diagnosis

Primary diagnosis	Hybrid	Video only	All TD
Acneiform	37 (14%)	593 (33%)	630 (31%)
Inflammatory	145 (54%)	874 (49%)	1019 (49%)
Lesion	85 (32%)	330 (18%)	415 (20%)
Total index encounters	267 (13%)	1797 (87%)	2064

Demonstrates trends in use of teledermatology models across diagnostic categories. Telephone visits, exclusively store-and-forward encounters, or incomplete visits were excluded. The average patient age was 40 years with sex distribution as 68.5% female and 31.5% male. The patient sample was 61% White, 18% Black, 8% Asian or Pacific Islander, 4% Hispanic or Latino, and 9% other or unknown.

Within the hybrid model, photos were uploaded for inflammatory (*N* = 520, 63%), followed by lesion-specific (*N* = 209, 25%), and acneiform conditions (*N* = 98, 12%). For hybrid encounters, a high rate of photo usability (95%) was similar to prior studies^5^ and a low rate of anatomically sensitive photos (5% groin/buttocks) was noted. The mean number of photos per encounter for inflammatory (3.41), acneiform (2.66), and lesion-specific (2.44) conditions were statistically different by one-way ANOVA (*P* < .01). By post hoc unpaired t-tests, inflammatory conditions averaged significantly more photos per encounter than lesions (*P* < .01), but other pairings did not differ significantly. Most photos featured a diagnosis assigned to the encounter (91%) versus a pregnancy test or other.

Nearly half of the uploaded photos were requested by the physician (46.8%), followed by patients (39.5%), and office staff (2.2%) or unknown initiators (9.7%) (Fig 1).Chi-squared analysis revealed that initiator and condition were statistically dependent (*P* = .004). Only for lesion-specific conditions did physicians more commonly request photos as compared to patients or staff/other. (30% vs 19%). This study is limited as a retrospective study comparing only 2 modalities of teledermatology within a single institution with a single Epic based platform during the pandemic.

**Fig 1:**
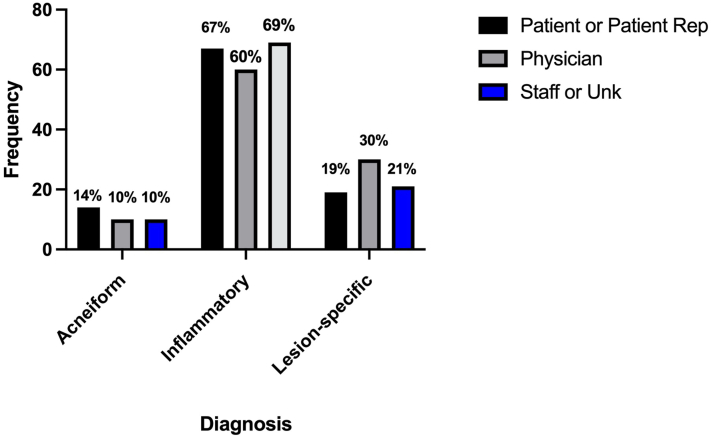
Skin diagnosis and photo uploading initiators. Proportion of images submitted by patients or their representatives, physicians, and staff or unknown sources across acneiform, inflammatory, and lesion-specific diagnoses in the hybrid teledermatology model. The electronic medical record workflow provided no standardized patient instructions regarding uploading photos through patient portal messages before or after a visit. Patients could upload 3 photos per message with the ability to send multiple messages in a hybrid encounter.

Within a teledermatology workflow that lacks standardized patient instruction, this study demonstrates an advantage of hybrid over video visits for lesion-specific conditions. Hybrid visits were used at a higher rate with physicians more frequently requesting photos for lesions as compared to other diagnoses. Inflammatory conditions demonstrated the highest photo upload rate per encounter. As a result, the hybrid model demonstrate higher utility in treating lesion-specific and inflammatory conditions via teledermatology while video-only visits suffice for acneiform conditions.

## Conflicts of interest

None disclosed.
